# Interferon-α Subtypes in an *Ex Vivo* Model of Acute HIV-1 Infection: Expression, Potency and Effector Mechanisms

**DOI:** 10.1371/journal.ppat.1005254

**Published:** 2015-11-03

**Authors:** Michael S. Harper, Kejun Guo, Kathrin Gibbert, Eric J. Lee, Stephanie M. Dillon, Bradley S. Barrett, Martin D. McCarter, Kim J. Hasenkrug, Ulf Dittmer, Cara C. Wilson, Mario L. Santiago

**Affiliations:** 1 Department of Medicine, University of Colorado Denver, Aurora, Colorado, United States of America; 2 Department of Immunology and Microbiology, University of Colorado Denver, Aurora, Colorado, United States of America; 3 Institute for Virology, University Hospital Essen, University of Duisburg-Essen, Essen, Germany; 4 Department of Surgery, University of Colorado Denver, Aurora, Colorado, United States of America; 5 Rocky Mountain Laboratories, National Institutes of Allergy and Infectious Diseases, National Institutes of Health, Hamilton, Montana, United States of America; Fred Hutchinson Cancer Research Center, UNITED STATES

## Abstract

HIV-1 is transmitted primarily across mucosal surfaces and rapidly spreads within the intestinal mucosa during acute infection. The type I interferons (IFNs) likely serve as a first line of defense, but the relative expression and antiviral properties of the 12 IFNα subtypes against HIV-1 infection of mucosal tissues remain unknown. Here, we evaluated the expression of all IFNα subtypes in HIV-1-exposed plasmacytoid dendritic cells by next-generation sequencing. We then determined the relative antiviral potency of each IFNα subtype *ex vivo* using the human intestinal Lamina Propria Aggregate Culture model. IFNα subtype transcripts from the centromeric half of the *IFNA* gene complex were highly expressed in pDCs following HIV-1 exposure. There was an inverse relationship between *IFNA* subtype expression and potency. IFNα8, IFNα6 and IFNα14 were the most potent in restricting HIV-1 infection. IFNα2, the clinically-approved subtype, and IFNα1 were both highly expressed but exhibited relatively weak antiviral activity. The relative potencies correlated with binding affinity to the type I IFN receptor and the induction levels of HIV-1 restriction factors Mx2 and Tetherin/BST-2 but not APOBEC3G, F and D. However, despite the lack of APOBEC3 transcriptional induction, the higher relative potency of IFNα8 and IFNα14 correlated with stronger inhibition of virion infectivity, which is linked to deaminase-independent APOBEC3 restriction activity. By contrast, both potent (IFNα8) and weak (IFNα1) subtypes significantly induced HIV-1 GG-to-AG hypermutation. The results unravel non-redundant functions of the IFNα subtypes against HIV-1 infection, with strong implications for HIV-1 mucosal immunity, viral evolution and IFNα-based functional cure strategies.

## Author Summary

The therapeutic potential of recombinant IFNα against HIV-1 infection has been explored for 25 years, but its effectiveness was inconsistent. However, these clinical trials administered IFNα2, which is only one member of a 12-protein family of IFNα subtypes. More recently, IFNα was found to activate ‘restriction factors’–proteins that can directly inhibit HIV-1. To date, it remains unknown which IFNα subtypes are produced by professional IFNα producing cells known as plasmacytoid dendritic cells and which IFNα subtypes are more effective in inhibiting HIV-1 infection in the gastrointestinal tract, the primary site of early HIV-1 replication. Here, we show that weaker IFNα subtypes were more highly expressed following HIV-1 infection. Using an infection platform that captures important characteristics of early HIV-1 infection in the gut, several IFNα subtypes were found to be more effective at inhibiting HIV-1 than IFNα2. In particular, IFNα8 and IFNα14 more potently reduced the infectivity of HIV-1 virions, an activity that can be attributed to the APOBEC3 proteins. Our findings strongly support the evaluation of potent IFNα subtypes in currently evolving HIV-1 curative strategies.

## Introduction

The type I interferons (IFNs) are critical players in the innate immune response against viral infections. Shortly after infection, these cytokines are rapidly induced, stimulating an antiviral state through the induction of hundreds of interferon-stimulated genes (ISGs) [1]. This family of cytokines include IFNα, the first cytokine produced through recombinant DNA technology and tested in clinical trials against many infectious diseases [2]. Notably, IFNα is a collective term for 12 unique IFNα proteins or subtypes expressed by 13 *IFNA* genes that are tandemly arrayed on human chromosome 9. However, most clinical trials only utilize recombinant IFNα2, the subtype that is currently licensed for the treatment of hepatitis B virus (HBV) and HCV infection. IFNα2 was also evaluated for reducing HIV-1 plasma viral loads during chronic infection. However, the variable levels of efficacy observed [3–6] and the advent of potent and safer antiretroviral drugs reduced enthusiasm for the use of IFNα in the clinical management HIV-1 infection. Two major developments in recent years renewed interest in IFNα as a therapeutic for HIV-1 infection: (1) the discovery of antiretroviral restriction factors, most of which are induced by IFNα [7]; and (2) the improved prospects in achieving functional HIV-1 cure, which may be advanced through IFNα-based therapies [8,9]. However, this renewed interest also raised unanswered questions on the basic biology of IFNα, including the biological consequences of having an expanded *IFNA* gene family [10,11]. In fact, the relative expression, antiviral potency and restriction factor mechanisms employed by the various IFNα subtypes against HIV-1 infection remains unclear.

One potential advantage for the expansion of the *IFNA* gene family could be the diversification of regulatory elements, which would allow the infected host to differentially express *IFNA* genes in response to diverse stimuli. Plasmacytoid dendritic cells (pDCs) are the primary producers of IFNα *in vivo* [12], and exposure of pDCs to HIV-1 or HIV-1 infected cells resulted in a dramatic rise in IFNα production [13,14]. Measurements of total IFNα proteins rely on antibodies that may have different binding affinities to the IFNα subtypes. Furthermore, antibodies that can distinguish the various IFNα subtypes are not yet available. IFNα expression is primarily regulated at the mRNA level [15]. Innate sensing of viruses, for example through Toll-like receptors (TLRs), results in a signaling cascade that leads to the activation and recruitment of transcription factors to the *IFNA* promoter(s) [16]. Thus, quantitative real-time PCR (qPCR) is a standard procedure used by many laboratories to measure *IFNA* gene expression, with increasing recognition on the importance of obtaining *IFNA* subtype distribution for understanding retroviral pathogenesis [17]. However, quantifying the expression of the different *IFNA* subtype genes is complicated by their high sequence homology (78 to 99%). Nevertheless, *IFNA* subtype expression profiles of pDCs were evaluated using quantitative real-time PCR assays developed for each *IFNA* gene [15,18–20]. Humanized mice exposed to TLR7 agonists showed prominent expression of *IFNA2* and *IFNA14* in pDCs [18] but other studies showed equal expression of all *IFNA* subtypes following TLR ligand stimulation [15,19]. These discrepancies suggested that measuring *IFNA* distribution by qPCR may be difficult to reproduce across laboratories. Moreover, performing 12 qPCR reactions for each *IFNA* subtype would not be ideal for limited biological samples. The lack of a robust method to quantify *IFNA* distribution is therefore a significant hurdle in understanding the role of *IFNA* subtypes in human health and disease.

Functional diversification may be another evolutionary advantage for an expanded IFNα gene family. Although all IFNα subtypes signal through the same type I interferon receptor (IFNAR), the IFNα subtypes exhibited different binding affinities for the IFNAR-1 and IFNAR-2 subunits [21,22]. This might result in different signaling pathways induced by IFNα subtypes [23] and in distinct expression patterns of ISGs *in vitro* [24]. *In vivo*, mouse IFNα subtypes exhibited different potencies against herpes simplex virus 1, murine cytomegalovirus, vesicular stomatitis virus (VSV), influenza virus and Friend retrovirus [11,25]. Altogether, the data indicate that the IFNα subtypes are not functionally redundant, raising the immediate question of which IFNα subtypes are most potent against HIV-1. An early study revealed that IFNα2 may be the most potent, but only 6 IFNα subtypes were evaluated against an X4-tropic, lab-adapted HIV-1 strain in the MT-2 T cell line [26], thereby raising issues regarding physiological relevance.

IFNα is induced very early during HIV-1 infection [27], and blocking IFNAR signaling in the SIV/rhesus macaque model resulted in higher viral loads and pathogenesis [28]. The impact of the early IFNα response against HIV-1 most likely manifests in the gut-associated lymphoid tissue (GALT), as it is the major site of early HIV-1 amplification and spread that leads to a massive depletion of CD4+ T cells [29,30]. Prior success in infecting gut lamina propria mononuclear cells (LPMCs) with HIV-1 [31] led to the development of the Lamina Propria Aggregate Culture (LPAC) model [32,33]. The LPAC model allows for the robust infection of primary gut CD4+ T cells with CCR5-tropic HIV-1 strains, subsequently leading to CD4+ T cell depletion. Importantly, this model allowed for HIV-1 infection studies without the confounding effects of non-physiologic T cell activation, as HIV-1 can efficiently infect gut CD4+ T cells without exogenous mitogens [29–31]. Thus, the LPAC model is an ideal *ex vivo* platform to evaluate the relative potency of the various IFNα subtypes against HIV-1.

Identifying the key effectors behind the anti-HIV-1 activity of IFNα could pave the way for the design of novel IFNα-based therapeutics. The APOBEC3 proteins (A3G, A3F, A3D and A3H), Tetherin/BST-2 and Mx2 were considered as *bona fide* HIV-1 restriction factors [7,34–37]. These factors were proposed as effectors of the IFNα treatment effect based on correlative studies using IFNα clinical trial data [38,39] as well as cell culture data [35,40–42]. However, their regulation by diverse IFNα subtypes in mucosal CD4+ T cells has not yet been explored. APOBEC3 and Tetherin are counteracted by the HIV-1 Vif and Vpu, respectively [7], but it is important to note that these interactions are saturable. Induction of APOBEC3 and Tetherin expression may undermine the antagonism due to Vif and Vpu by offsetting the balance of these respective interactions. Tetherin and Mx2 inhibit HIV-1 in the infected cell, leading to a reduction in virus release [34–37]. In contrast, the APOBEC3 proteins are packaged into budding HIV-1 particles and inhibit replication in the next target cell by impeding reverse transcription and hypermutating reverse transcripts [43,44]. Thus, a strong case for APOBEC3 activity could be made if reduced HIV-1 virion infectivity and increased G→A hypermutation were both detected. We previously showed that treatment of Friend retrovirus-infected wild-type mice with IFNα reduced viral loads, but not in Apobec3 knock-out (KO) mice [45]. Given the longstanding evolutionary conflict between mammalian hosts and retroviruses [46], we hypothesized that the human APOBEC3 proteins may also act as effectors of IFNα treatment against HIV-1 in mucosal CD4+ T cells.

Here, we modeled the role of the IFNα subtypes during acute HIV-1 infection. Using a novel next-generation sequencing-based method, we quantified the relative expression of the IFNα subtypes following HIV-1 exposure in pDCs, and determined the relative antiviral potency of each IFNα subtype in the LPAC model. Moreover, we determined the induction profiles of known HIV-1 restriction factors following treatment with individual IFNα subtypes, and provide evidence that the APOBEC3 proteins may serve as key effectors for the antiviral activity of IFNα against HIV-1.

## Results

### *IFNA* subtype expression in HIV-1-exposed pDCs is linked to chromosomal position

**Fig 1:**
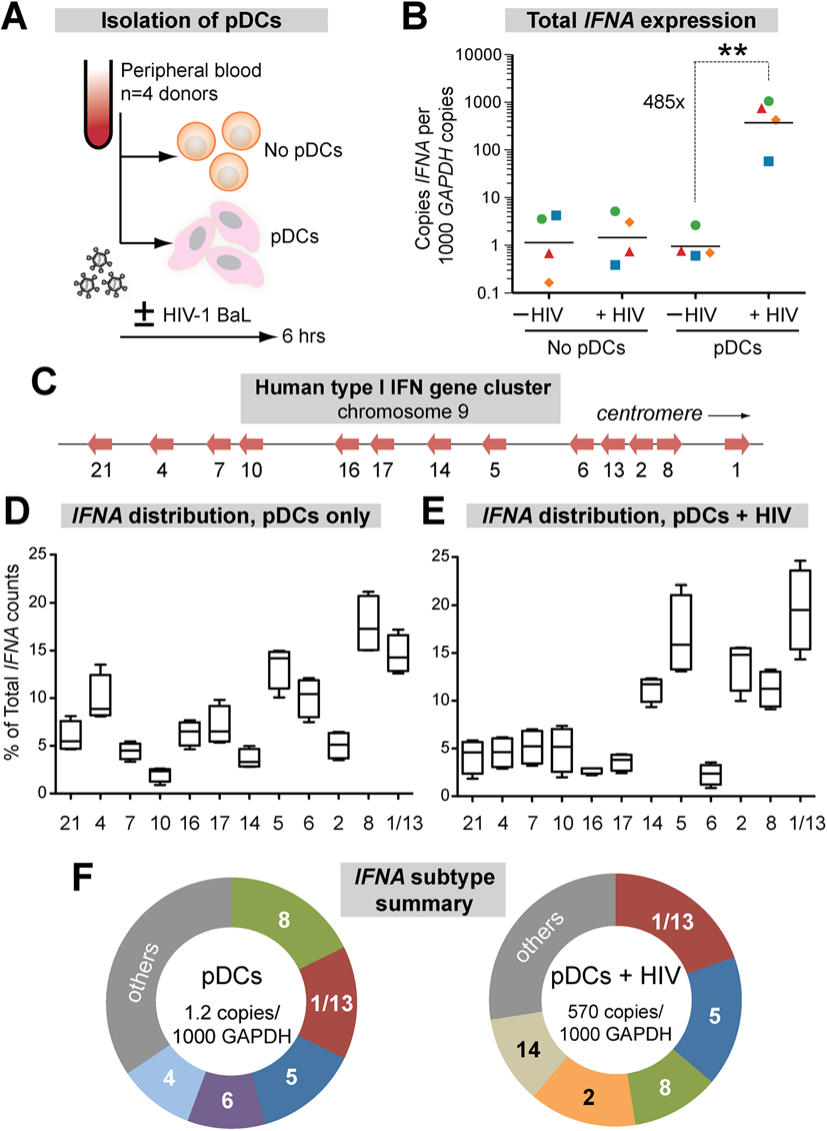
Expression of IFNα subtypes in pDCs following HIV-1 exposure. (A) Isolation pDCs. pDCs were enriched by negative selection from PBMCs of healthy donors (n = 4). Both pDC-enriched and pDC-negative fractions were exposed to 250 ng p24 of HIV-1_BaL_ by 2 hr spinoculation at room temperature and incubated 4 hr at 37°C. (B) Total copies of *IFNA* by qPCR normalized to 10^3^ copies *GAPDH*. Each color/shape combination corresponds to one donor. Data were analyzed using a 2-tailed Student’s paired t-test. **, *p*<0.01. (C) Type I IFN gene cluster in human chromosome 9. (D, E) Percentage of total *IFNA* sequence counts for each *IFNA* subtype in (D) Mock or (E) HIV-1_BaL_ infection. *IFNA* subtypes on the x-axis were shown relative to chromosomal position. The values for *IFNA1/13* were presented at the genomic position for *IFNA1*. Box-and-whisker plots correspond to 25-75^th^ percentiles with bars corresponding to minimum and maximum values. Median values were indicated as solid lines within the boxes. (F) Relative abundance of the 5 predominant subtypes expressed in pDCs ± HIV-1 exposure.

Plasmacytoid DCs (pDCs) are the primary sources of IFNα *in vivo*, migrating to the GALT from the periphery during acute SIV infection [47] and accumulating in mucosal tissues during chronic HIV-1 infection stages [48,49]. To date, the IFNα subtypes produced by pDCs following HIV-1 sensing remain unknown. To determine the expression levels of each IFNα subtype, we designed 2 complementary assays using primers designed in the most conserved regions of the 13 *IFNA* genes (S1 Fig). Using these primers, total *IFNA* expression relative to the housekeeping gene *GAPDH* could be measured by qPCR, whereas *IFNA* subtype distribution could be quantified by next-generation sequencing. We used negative selection to enrich pDCs from PBMCs from 4 healthy donors and exposed the cells to HIV-1 virions (R5-tropic BaL strain) for 6 hrs (Fig 1A). A 6 hr timepoint was chosen to ensure the viability of the pDCs, which significantly decline by 24 h post-culture [50], while capturing the initial burst of *IFNA* expression following viral sensing. Total *IFNA* expression was induced 485-fold in pDCs following HIV-1 exposure, but not in PBMCs lacking pDCs, confirming that pDCs are the main producers of IFNα (Fig 1B).

We next quantified the relative abundance of each IFNα subtype in pDCs ± HIV-1. Primers in the conserved regions were modified with Illumina-sequencing adaptors, and the *IFNA* subtype designation for each sequence was determined based on the polymorphic regions in the amplicon. *IFNA1* and *IFNA13* encode identical proteins and had identical DNA sequences in the region amplified, so these genes were counted together as *IFNA1/13*. On average, 9,543 *IFNA* sequence reads were analyzed per donor per condition. The *IFNA* genes were aligned according to their relative genomic positions and their proportional expression values are shown (Fig 1C).

The proportional expression of different *IFNA* subtypes by pDCs from different donors was very consistent both in naïve cultures (Fig 1D) and following HIV-1 exposure (Fig 1E). Interestingly, there was a strong bias towards expression of *IFNA* genes at the centromeric half of the *IFNA* complex following HIV-1 exposure (Fig 1E). Five out of six *IFNA* genes in this genomic cluster accounted for >70% of the *IFNA* subtypes expressed by pDCs following HIV-1 exposure (Fig 1F). The exception was *IFNA6*, which decreased as a percentage of the total *IFNA*. The augmented *IFNA* subtype expression levels were independent of genomic orientation, as *IFNA2* and *IFNA8* were both highly expressed yet had opposite genomic orientations (Fig 1C). We then determined the absolute copy numbers of each *IFNA* subtype by multiplying the percentage values (Fig 1D and 1E) with the total copy numbers (Fig 1B). The absolute copy numbers of all *IFNA* subtypes increased in pDCs following HIV-1 exposure, though to varying degrees (S2 Fig). *IFNA14*, *IFNA2* and *IFNA10* were induced over 1000-fold following HIV-1 exposure of pDCs, whereas *IFNA6* was induced by ~100-fold. Overall, the results revealed a pattern of *IFNA* gene induction after HIV-1 exposure that appeared to be linked to chromosomal position.

### IFNα subtypes differentially inhibit HIV-1 replication in the LPAC model

**Fig 2:**
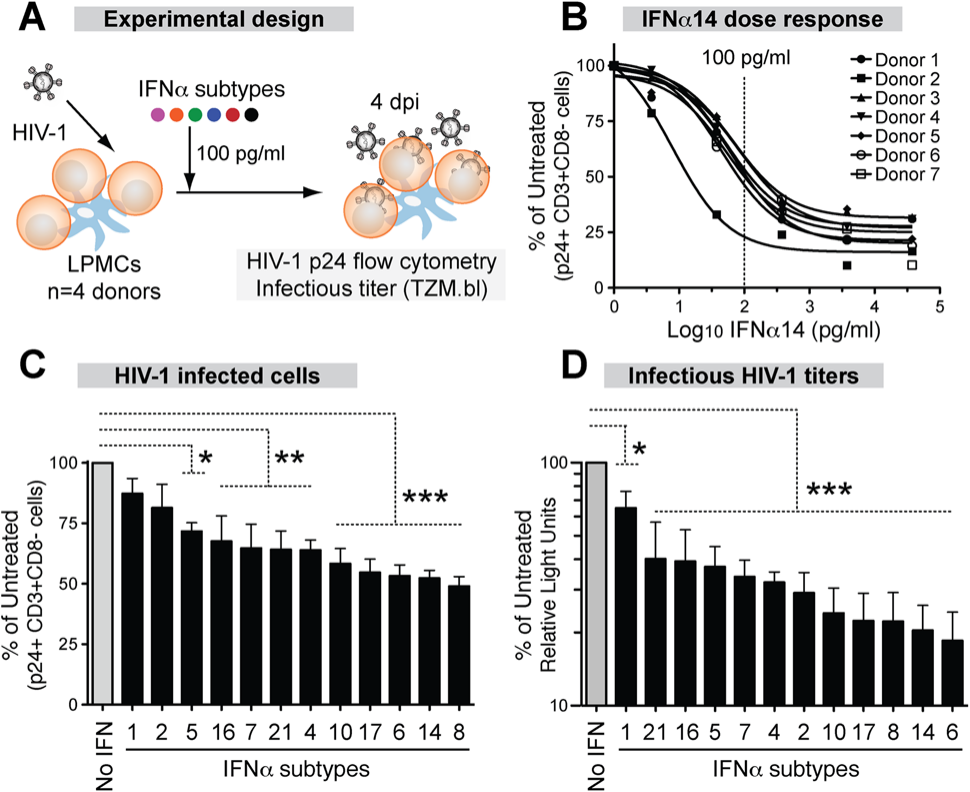
Inhibition of HIV-1 by 12 IFNα subtypes in the LPAC model. (A) LPMCs (n = 4 donors) were infected with HIV-1_BaL_ (10 ng p24/10^6^ cells) by spinoculation for 2 hrs. Each IFNα subtype was added individually at 100 pg/ml, and cells were harvested at 4dpi. (B) Dose-response curve of IFNα14 for inhibition of HIV-1 infection (p24+CD3+/CD8- lymphocytes). Vertical dashed line indicates the IFNα dose used for subsequent experiments. Inhibition of (C) cellular HIV-1 infection and (D) infectious titer, normalized to untreated samples. Bars correspond to the means with SEM error bars from 4 LPMC donors. The x-axis was arranged from the least to the most potent IFNα subtype. Repeated measures ANOVA with Dunnett’s multiple comparison test was performed on raw infection values. Pairwise comparisons were each against the no IFNα control. ns, not significant at *p*>0.05; *, *p*<0.05; **, *p*<0.01; ***, *p*<0.001.

Since the GALT is the major site of early HIV-1 amplification and spread, we utilized LPAC as a physiologically relevant model to determine the relative anti-HIV potency of each IFNα subtype. In particular, we were interested in whether IFNα2, the subtype approved for clinical use, was the optimal IFNα subtype for inhibiting HIV-1. Fig 2A outlines the experimental infection protocol.

Analyzing the HIV-1 potency of all 12 IFNα subtypes at multiple doses was not feasible in the LPAC model because of the limited number of LPMCs available per donor. Thus, initial dose-response tests were performed with IFNα14, which potently inhibited HIV-1 in a pilot experiment. Following infection with HIV-1_BaL_, LPMCs were rinsed with culture media and resuspended to various IFNα14 concentrations. Infection levels were evaluated at 4 days post-infection (dpi) to capture not only the impact of restriction factors that inhibit HIV-1 virus production, but also those that inhibit virion infectivity, which would decrease infection after one round of replication (S3 Fig). The percentage of infected CD4+ T cells was measured by detecting intracellular HIV-1 p24 capsid expression by flow cytometry, as we previously described [32,33]. To account for HIV-1 Nef and Vpu-mediated CD4 downregulation [51], we gated on CD3+CD8- cells. A screen of LPMCs from 7 donors revealed that IFNα14 restricted productive HIV-1 infection, and that the inhibition was saturable at higher concentrations (Fig 2B). The majority of the LPMC donors had similar sensitivity to IFNα14-treatment, with the exception of one donor who responded to lower concentrations. An IFNα concentration of 100 pg/ml was in the linear range of the dose response curve (~50% inhibition), and was chosen for the subsequent evaluation of all IFNα subtypes in 4 LPMC donors. This concentration was also within the range of IFNα levels in plasma following HIV-1 infection *in vivo* [52]. Majority of the cells in the LPMC donors used were CD3+ T cells (88% ± 3%). On average, 65% of the LP T cells were CD4+. Myeloid DCs and gamma-delta T cells account for <1% of the total LPMC subpopulations, respectively.

Recombinant IFNα subtypes were added to LPMCs (100 pg/ml) after spinoculation (Fig 2A). At 4 dpi, HIV-1 infected cells were quantified by detecting intracellular p24 by flow cytometry as in Fig 2B. There were clear differences in the potency of the IFNα subtypes in inhibiting productive HIV-1 infection (Fig 2C). IFNα8, IFNα14 and IFNα6 showed the highest levels of inhibition, whereas IFNα1 and IFNα2 had no significant effect. The supernatants were also tested for infectious HIV-1 titers using the TZM.bl assay (S3 Fig). Again, the same 3 IFNα subtypes were most potent, whereas IFNα1 remained the least potent (Fig 2D). The antiviral potency of the different IFNα subtypes as measured by p24 flow cytometry and the TZM.bl assay significantly correlated with each other (S4A Fig). Although IFNα2 had no significant effect on cellular HIV-1 infection (Fig 2C), it moderately reduced infectious titers (Fig 2D). Overall, the LPAC data revealed differences in the potencies of IFNα subtypes in inhibiting HIV-1 infection. IFNα2, the current subtype approved for clinical use, was one of the least potent subtypes.

### HIV-1-exposed pDCs express high levels of IFNα subtypes with low antiviral activity

**Fig 3:**
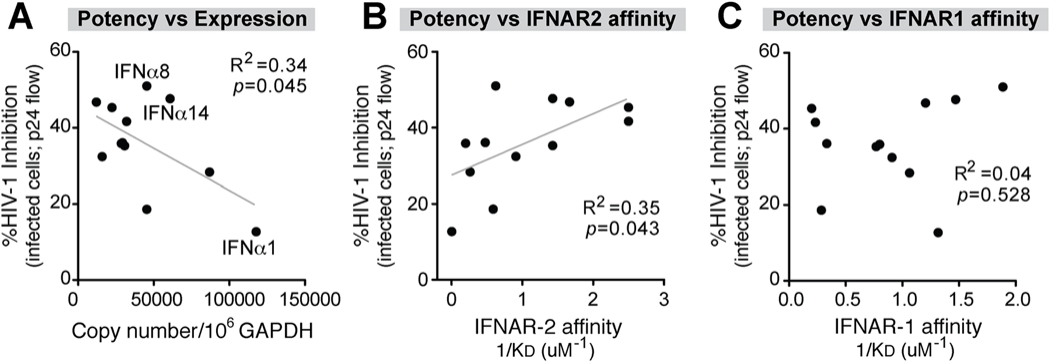
Correlation between IFNα antiviral potency and various parameters. Percent cellular HIV-1 inhibition values relative to no IFNα treatment control were obtained from Fig 2C. (A) Correlation between IFNα potency and absolute *IFNA* subtype copy numbers, based on multiplying % *IFNA* distribution in Fig 1D and total *IFNA* copies in Fig 1B. IFNα subtype potencies were also correlated with non-log-transformed binding affinity (1/K_D_ or K_A_) to (B) IFNAR-2 and (C) IFNAR-1 based on published data [22]. For all panels, Pearson correlation analysis was performed, with R^2^ values and *p*-values shown. Best-fit linear regression curves are shown in the correlation was significant (*p*<0.05).

To investigate whether the IFNα response of pDCs following HIV-1 exposure was biased towards the expression of the most potent antiviral IFNα subtypes, we next determined the relationship between IFNα subtype expression levels and relative potency. Absolute *IFNA* subtype copy numbers were calculated by multiplying the total *IFNA* copies (Fig 1B) by the percentage of total *IFNA* for each subtype (Fig 1E). This provided an estimated copy number of each *IFNA* subtype per 10^6^ copies of *GAPDH*. Using these values, a significant inverse correlation was observed between *IFNA* subtype expression and potency (Fig 3A and S4B Fig). This correlation can be exemplified as follows. IFNα1 was highly expressed but ineffective at inhibiting HIV-1 replication. IFNα6, one of the most potent subtypes, was among the least abundant following HIV-1 exposure. IFNα2 showed a very high fold-increase following HIV-1 exposure relative to baseline but had weak antiviral efficacy. IFNα5 is expressed at higher relative abundance (Fig 1F) but was also weakly antiviral. These results revealed that the predominant *IFNA* subtypes produced by pDCs following HIV-1 exposure had low antiviral potency. Two notable exceptions were IFNα8 and IFNα14, which exhibited strong anti-HIV-1 activity and also had high expression in pDCs following HIV-1 exposure (Fig 3A). Exclusion of the IFNα8 and IFNα14 datapoints further strengthen the inverse correlation (R^2^ = 0.62, *p* = 0.007).

Data from the Schreiber group [22] revealed that different IFNα subtypes exhibited variable binding affinities to IFNAR as estimated by surface plasmon resonance against each subunit, IFNAR-1 and IFNAR-2. We therefore determined if IFNα subtype anti-HIV-1 potency (Fig 2C and 2D) correlated with published binding affinity data to IFNAR [22]. There was a significant positive correlation between antiviral potency and binding affinity (K_A_) to IFNAR-2 (Fig 3B and S4C Fig), but not the IFNAR-1 subunit (Fig 3C and S4D Fig). These analyses suggested that following HIV-1 exposure, pDCs produced IFNα subtypes with relatively low antiviral activity and lower binding affinity to IFNAR-2. In particular, IFNα1 was expressed at high levels by pDCs exposed to HIV-1 virions but had the weakest IFNAR-2 binding affinity and the lowest anti-HIV-1 potency in the LPAC model.

### Differential induction of antiretroviral ISGs by IFNα subtypes

**Fig 4:**
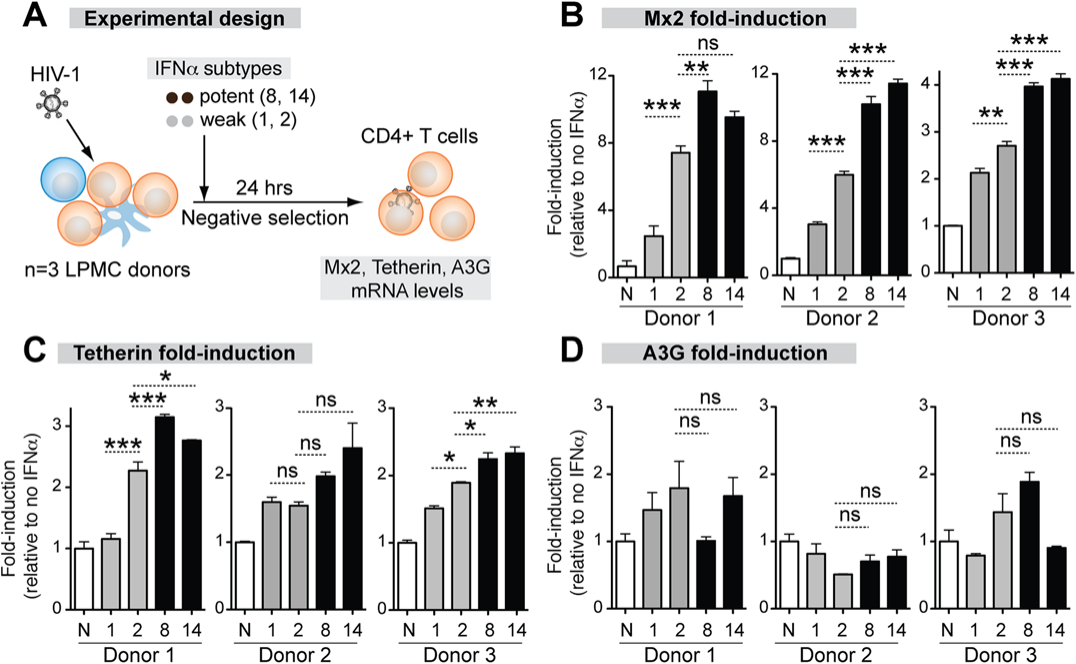
Correlation between ISG induction and IFNα subtype antiviral potency. (A) LPMCs (n = 3 donors) were thawed and infected with HIV-1_BaL_, then treated with 100 pg/ml of weak (gray bars) and potent (black bars) IFNα subtypes. IFNα1, IFNα2, IFNα8 and IFNα14 shown simply as 1, 2, 8 and 14. After 24 hr, CD4+ T cells were negatively selected and RNA extracted for qPCR. ISGs were quantified using Taqman qPCR normalized to GAPDH levels. Mean fold-induction values and SEM error bars for (B) Mx2, (C) Tetherin/BST-2 and (D) A3G relative to no IFNα control (N) are shown for the 3 donors. Data were analyzed using repeated measures ANOVA with Dunnett’s multiple comparison test. Statistical support for differences in fold-induction between IFNα1 and IFNα2, and IFNα2 and IFNα8/14 are shown. ns, not significant at *p*>0.05; *, *p*<0.05; **, *p*<0.01; ***, *p*<0.001.

The correlation between antiviral potency and IFNAR binding affinity suggested that the more potent IFNα subtypes might trigger higher ISG induction. To test this hypothesis, we quantified the mRNA expression levels of the IFNα-inducible HIV-1 restriction factors Mx2, Tetherin and APOBEC3 in LP CD4+ T cells after stimulation with representative IFNα subtypes. We focused on CD4+ T cells, the major cellular targets of HIV-1 replication in the GALT, but not intestinal macrophages, which are non-permissive to HIV-1 infection [53]. We selected IFNα8 and IFNα14 as potent IFNα subtypes due to their high affinity, highest antiviral potency in the LPAC model and high expression level in pDCs. IFNα1 and IFNα2 were selected as weak IFNα subtypes due to their relatively low affinity, weaker antiviral activity in the LPAC model (with IFNα2 being more potent than IFNα1), but high expression level in HIV-1-exposed pDCs (IFNα1 and IFNα2). IFNα2 was also chosen because of its clinical relevance. LPMCs were infected with HIV-1_BaL_ and 100 pg/ml IFNα was administered. After 24 hr, CD4+ T cells were negatively selected and ISG mRNA expression was evaluated by qPCR (Fig 4A).

The magnitude of ISG induction was donor-dependent so the data for each donor are presented. (Fig 4B to 4E). The ISG expression that best correlated with the relative antiviral activities of the IFNα subtypes was Mx2 (Fig 4B). IFNα8 (3 of 3 donors) and IFNα14 (2 of 3 donors) more significantly induced Mx2 compared to IFNα1 and IFNα2. IFNα2, which showed moderate antiviral activity (Fig 2D), more significantly induced Mx2 compared to IFNα1 in 3 of 3 donors. Tetherin induction exhibited trends similar to Mx2, but the differences were not as consistent between donors (Fig 4C and 4D). Overall, the more antiviral IFNα subtypes induced Mx2 and Tetherin to higher levels. In contrast, A3G (Fig 4E) was not significantly induced by any of the IFNα subtypes. A3F and A3D expression were induced in a few cases with IFNα treatment (S6 Fig), but the induction levels did not correlate with the relative anti-HIV-1 potency of the IFNα subtypes.

### Potent IFNα subtypes inhibit HIV-1 virion infectivity

We previously demonstrated that mouse Apobec3 was the primary effector of IFNα treatment against Friend retrovirus infection despite not being transcriptionally induced [45]. We therefore investigated the potential contribution of human APOBEC3 proteins to the IFNα-treatment effect. The APOBEC3 proteins A3G, A3F, A3D and A3H do not inhibit HIV-1 in the producer cell. Instead, these proteins get packaged into HIV-1 virions and inhibit replication in the next target cell. Thus, non-infectious virion release is a distinguishing feature of APOBEC3-mediated retrovirus restriction [54,55]. By contrast, most restriction factors such as Mx2 and tetherin inhibit virus particle production in the infected cell [7]. Virion infectivity is typically measured by determining the ratio of infectious titer as measured by the TZM.bl assay and the total viral particles released in the supernatant as measured by HIV-1 p24 ELISA (S3 Fig).

**Fig 5:**
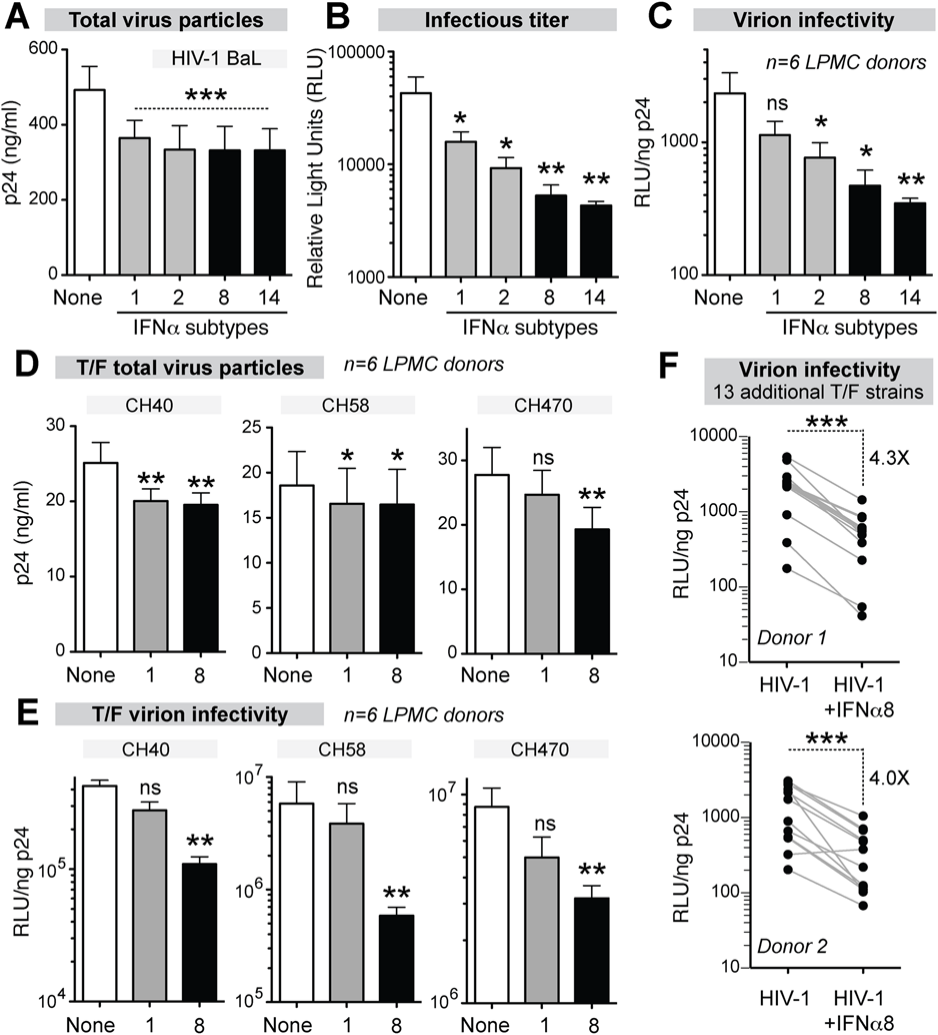
Potent IFNα subtypes inhibit HIV-1 virion infectivity. LPMCs (n = 6 donors) were infected with HIV-1_BaL_, treated with IFNα subtypes with weak (gray bars, IFNα1 and IFNα2) or strong (black bars, IFNα8 and IFNα14) antiviral activity. Supernatants at 4 dpi were evaluated for (A) virus particle titer by p24 ELISA, (B) infectious titer by TZM.bl assay and (C) virion infectivity using the ratio of values from (A) and (B). Similar analyses were performed for HIV-1 T/F strains CH40, CH58 and CH470 with IFNα1 and IFNα8, with data on (D) virus particle titer and (e) virion infectivity shown. In (A-E), bars correspond to means with SEM error bars and statistical analyses are shown for comparisons between IFNα-subtype treated samples and no IFNα control (‘none’). Data were analyzed using repeated measures ANOVA with Dunnett’s multiple comparison test. In (E), the data were analyzed using Friedman’s test to account for non-Gaussian distribution, followed by Dunn’s posthoc analysis. (F) Thirteen additional T/F HIV-1 strains (AD17, CH106, CH607, REJO, RHPA, THRO, STCOr1, STCOr2, WARO, MCST, RHGA, TRJO and WITO) were incubated with or without IFNα8 after infecting LPMCs from 2 donors. At 4 dpi, virion infectivities were computed as in (C) and (E). Each connected line corresponds to a T/F HIV-1 strain. Data were analyzed using a 2-tailed paired Student’s t-test. For all panels, ns, not significant at *p*>0.05; *, *p*<0.05; **, *p*<0.01; ***, *p*<0.001.

LPMCs from 6 donors were infected with HIV-1_BaL_ and were treated with IFNα1, IFNα2, IFNα8 and IFNα14. At 4 dpi, all 4 IFNα subtypes inhibited virus particle release to the same extent (Fig 5A). By contrast, the infectious titers were reduced significantly more by IFNα8 and IFNα14 compared to IFNα1 and IFNα2 (Fig 5B). Thus, inhibition of virion infectivity correlated with the antiviral efficacy of the IFNα subtypes (Fig 5C). In particular, IFNα8 and IFNα14 were the most potent at inhibiting virion infectivity whereas IFNα1 had no significant effect. In order to confirm that the findings were not specific to HIV-1_BaL_, LPMCs were infected with transmitted/founder (T/F) HIV-1 strains, which are infectious molecular clones reconstructed from acute HIV-1 infection samples [56–58]. In 6 LPMC donors, the antiretroviral activity of IFNα1 and IFNα8 against the T/F HIV-1 strains CH470, CH40, and CH58 were compared. IFNα1 and IFNα8 inhibited virus particle release to similar extents for CH40 and CH58 (Fig 5D), whereas CH470 particle release was slightly more inhibited by IFNα8. In virion infectivity assays, IFNα8 more potently inhibited the 3 T/F HIV-1 strains (Fig 5E). We also evaluated the impact of IFNα8 in 13 additional T/F HIV-1 strains in 2 LPMC donors. IFNα8 treatment resulted in a highly significant (~4-fold) decrease in virion infectivity (Fig 5F). IFNα14 treatment also significantly inhibited the virion infectivity of these T/F HIV-1 strains (S5 Fig). These data indirectly suggested that the more potent IFNα subtypes augmented APOBEC3-mediated restriction of multiple HIV-1 strains.

### IFNα8 and IFNα1 treatment promotes APOBEC3G-mediated HIV-1 hypermutation

**Fig 6:**
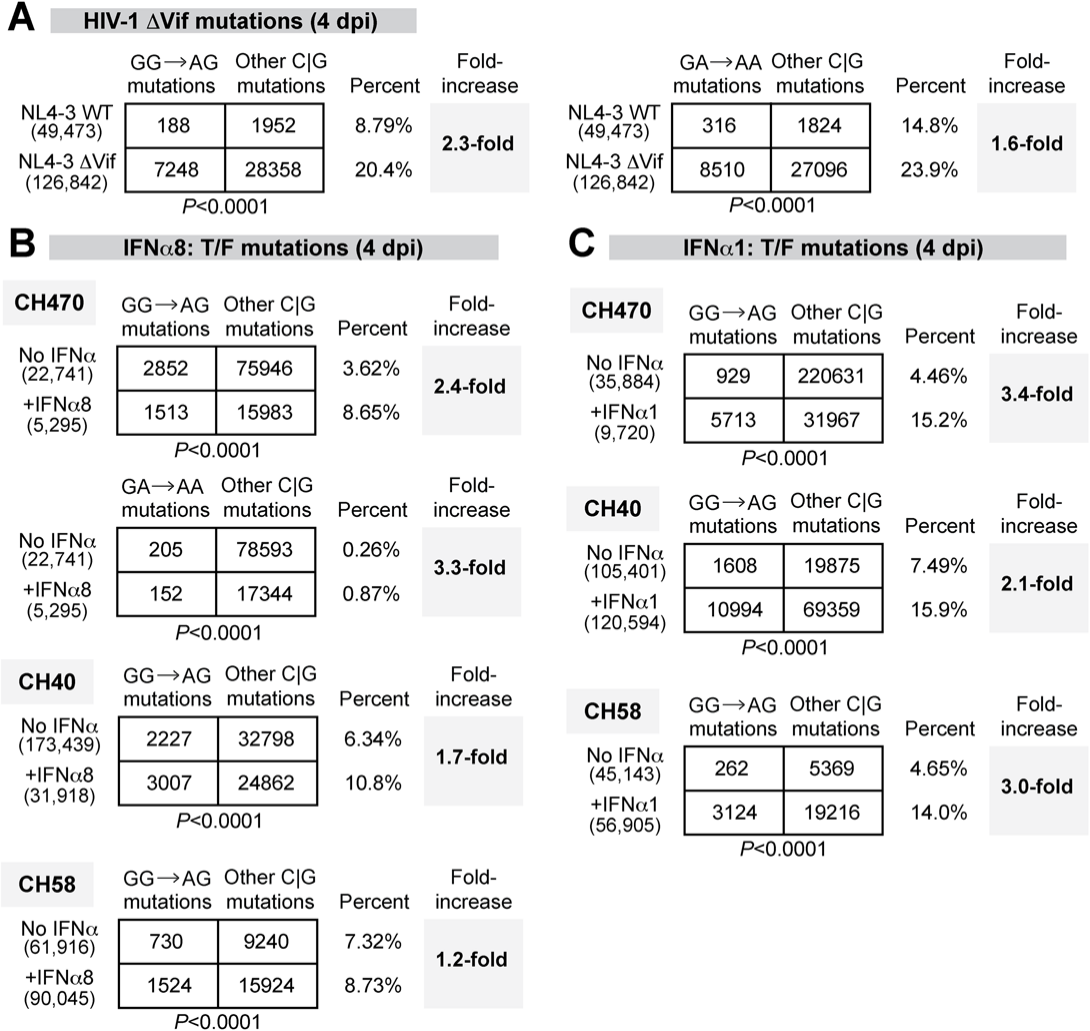
Potent and weak IFNα subtypes enhanced APOBEC3-mediated hypermutation against multiple HIV-1 strains. G-to-A mutation rates were estimated for (A) HIV-1 NL4-3 WT versus ΔVif infected LPMCs at 4 dpi; T/F strains infected LPMCs with or without (B) IFNα8 or (C) IFNα1 (100 pg/ml) treatment at 4 dpi. For all panels, DNA was extracted from HIV-1 infected cells or tissues and a 420–450 bp HIV-1 gp41/nef region was amplified using barcoded Illumina primers. Each sequence analyzed was represented at least twice per donor. Sequence reads from multiple donors were pooled for each virus condition. The number of sequence reads analyzed was shown in parentheses. The percentage of the respective mutations relative to the total number of C or G mutations were shown, and the fold-increase relative to the (A) WT or (B, C) no-treatment control were shown in bold. Differences in the proportions of GG→AG or GG→AA mutations relative to other C or G mutations between the treatment groups were analyzed using a 2×2 contingency test with Yates’ correction.

The APOBEC3 proteins A3F, A3D and A3H mutated HIV-1 reverse transcripts with a preferred TC context, leading to GA→AA mutations in the retroviral plus strand, whereas A3G preferentially mutated in the CC context, leading to proviral GG→AG mutations [59]. Thus, the magnitude of retroviral mutations in the GA→AA versus GG→AG context could be used to determine the APOBEC3 members responsible for HIV-1 G-to-A hypermutation and to provide additional evidence of APOBEC3 involvement in HIV-1 restriction. To quantify APOBEC3-mediated retroviral mutations, we recently developed a next-generation sequencing approach to quantify mouse retrovirus hypermutation [60]. To extend this method to HIV-1, we designed barcoded Illumina primers encompassing gp41/nef (420–450 bp depending on the strain), a region that may be more susceptible to APOBEC3-mediated deamination due to longer retention in single-stranded form during reverse transcription [61]. We initially tested the method by infecting LPMCs with WT HIV-1 NL4-3 and NL4-3ΔVif, which cannot counteract the effects of APOBEC3. The percentage of GG→AG and GA→AA mutations were computed against the mutations at C or G bases, which are directly modified by deaminases. As expected, there was a significant increase in GG→AG and GA→AA mutations in NL4-3ΔVif compared to WT at 4 dpi (Fig 6A). Thus, A3G and A3F/A3D/A3H actively mutated HIV-1ΔVif in gut CD4+ T cells.

Following the validation of the next-generation sequencing method, we next analyzed proviral HIV-1 sequences for evidence of GG→GA and GA→AA mutations following treatment with IFNα8 or IFNα1. LPMCs were infected with T/F HIV-1 strains CH470, CH40, and CH58. These strains were derived from infectious molecular clones and therefore allow for straightforward mutational analysis. These 3 HIV-1 strains also had reduced virion infectivity following IFNα8 but not IFNα1 treatment (Fig 5E). Untreated and IFNα-treated infected cells were harvested at 4 dpi. Sequences were pooled for each of the HIV-1 CH470, CH40, and CH58 strains, respectively, to allow for a thorough analysis of mutational patterns. A 2×2 contingency analysis was performed to test if IFNα had any effect on A3F/D/H-type (GA→AA) or A3G-type mutations (GG→AG) relative to the total number of C or G mutations. Following IFNα8 treatment, both GG→AG and GA→AA mutations significantly increased in CH470 (Fig 6B). GG→AG mutations also significantly increased in CH40, and to a lesser extent in CH58 (Fig 6B). Surprisingly, IFNα1 treatment also increased GG→AG mutations in CH40, CH58 and CH470 (Fig 6C). Thus, both IFNα8 and IFNα1 treatment increased proviral DNA mutations that were associated with A3G deaminase activity.

## Discussion

Acute HIV-1 infection is characterized by extensive virus replication in the GALT, suggesting that the innate immune response could have a considerable impact on early HIV-1 spread in this compartment. In particular, IFNα exhibited potent anti-HIV-1 properties *in vitro* and was one of the first cytokines induced during acute HIV-1 infection [27]. Blocking type I IFN signaling in the SIV/rhesus macaque model resulted in more severe pathogenesis [28]. T/F HIV-1 strains exhibited higher resistance to type I IFNs than counterpart chronic strains, suggesting that type I IFNs exerted a strong selective pressure during acute HIV-1 infection [57,58]. These studies suggested that the initial IFNα response may serve as a roadblock for HIV-1 replication and spread in the GALT. However, there were 12 IFNα subtypes, and to date, it remained unknown which IFNα subtypes were produced by pDCs, the professional IFNα-producing cells that rapidly migrate and reside in the GALT following HIV-1/SIV infection [47–49]. Moreover, only one subtype, IFNα2, was evaluated in clinical trials to reduce HIV-1 viremia. In fact, the clinical use of IFNα2 was largely driven by its status as the first IFNα subtype cloned for large-scale production [2], and not from a systematic evaluation of antiviral potencies in physiologically-relevant target cells. Thus, the current study was undertaken to investigate the relative expression of the different IFNα subtypes in pDCs and their antiviral potency in the LPAC model.

A major finding from this work was that IFNα8, IFNα6 and IFNα14 were the most effective at inhibiting HIV-1 replication in gut CD4+ T cells. By contrast, the antiviral activity of IFNα2 was weak at best. IFNα8, IFNα6, and IFNα14 exhibited strong binding affinities to IFNAR-2 [22]. This suggests that binding affinity to IFNAR-2, proposed as the first IFNAR subunit that binds IFNα [62], may contribute to the differential potencies of the IFNα subtypes. This notion was corroborated by the higher ISG induction profile for IFNα8 and IFNα14 compared to IFNα2. Sequence analyses of IFNα8, IFNα6 and IFNα14 in human populations revealed that DNA polymorphisms in these subtypes tend to preserve the amino acid sequence (e.g., purifying selection) [63], suggesting that these IFNα subtypes may have essential roles *in vivo*. Moreover, IFNα8 exhibited strong antiviral activity against other viruses [64]. Interestingly, using a novel method to quantify *IFNA* subtype distribution, we observed an inverse correlation between IFNα subtype expression in HIV-1-exposed pDCs and anti-HIV-1 potency. IFNα6 fit this trend–it was one of the least expressed IFNα subtypes in HIV-1-exposed pDC cultures. IFNα6 was also weakly expressed by pDCs stimulated with TLR ligands [15]. However, IFNα8 and IFNα14 were both potent and more abundantly produced by pDCs exposed to HIV-1. IFNα8 and IFNα14 were encoded within the centromeric half of the *IFNA* complex, suggesting that epigenetic mechanisms may regulate their expression. The data suggest that IFNα8 and IFNα14 may constitute the most potent antiviral fraction of the initial IFNα response against HIV-1 infection. However, it should be noted that IFNα8 and IFNα14 only account for ~20% of the total *IFNA* transcripts produced by pDCs following HIV-1 exposure.

The majority of the IFNα subtypes expressed by pDCs following HIV-1 exposure had relatively weak antiviral activity (*IFNA1*, *2* and 5 account for >40% of *IFNA* transcripts). In particular, the most expressed IFNα subtype, IFNα1, had the weakest antiviral activity. IFNα1 also exhibited very weak activity against VSV and HCV, and the lowest binding affinity for IFNAR-2 [22,64–66]. IFNα2 was also highly induced in pDCs post-HIV-1 exposure, consistent with another study showing IFNα2 was upregulated in HIV-1-infected individuals [67]. We speculate that IFNα1 and IFNα2 induction may be a strategy used by HIV-1 to evade a more potent IFNα response. However, the rationale for why humans evolved weakly antiviral IFNα subtypes in the first place remains unknown. One possibility is that weakly antiviral IFNα subtypes may be better at modulating other immunological processes. If true, then these IFNα subtypes could potentially elicit more adverse effects if administered therapeutically. IFNα2 therapy was long known to have undesirable clinical side-effects including fever, fatigue and lymphopenia [2]. Moreover, in an intriguing paradox, high IFNα expression levels during chronic HIV-1 infection correlated with disease progression [52,68]. This led some to propose blocking IFNα signaling in chronic HIV-1-infected individuals to reduce immune activation [69]. However, the IFNα subtypes responsible for the link between IFNα and chronic immune activation remains unknown. The development of the *IFNA* subtyping method described here should facilitate revisiting this phenomenon. In addition, further studies would be required to evaluate the tolerability profile of IFNα8, IFNα6 and IFNα14 relative to IFNα2 and IFNα1.

One possible strategy to harness the antiviral properties of IFNα for the design of safer HIV-1 therapeutics is to focus on its downstream antiviral effectors. Many ISGs were reported to have inhibitory activity against HIV-1 *in vitro* [70], but transcriptional induction levels may not predict the most potent antiviral effectors of IFNα [45]. In this study, the more antiviral IFNα subtypes induced Mx2 and Tetherin to a greater extent. Mx2 and Tetherin act on the producer cell, decreasing viral production. Thus, if the IFNα subtypes were acting through these restriction factors to inhibit HIV-1 replication, we would expect higher inhibition of virus production by the more potent IFNα subtypes. Surprisingly, this was not the case: IFNα1 inhibited virus particle production to a similar extent as IFNα8 and IFNα14. Thus, Mx2 or Tetherin may not be mediating the differences in antiviral potencies between the IFNα subtypes. In other words, the differential induction of Mx2 and Tetherin expression by potent versus weak IFNα subtypes may just reflect the magnitude of IFNAR signaling and not necessarily indicate the mobilization of these effector mechanisms.

The IFNα subtypes did not significantly upregulate A3G, A3F and A3D transcription in gut CD4+ T cells, consistent with previous data using IFNα in PBMCs [71,72]. Nonetheless, the relative potencies of the IFNα subtypes were associated with reduced virion infectivity, thus pointing to the APOBEC3 proteins as a significant antiviral effector of IFNα. The notion that the APOBEC3 proteins could act as significant effectors of potent IFNα subtypes makes evolutionary sense based on our studies in mice [45]. However, the mechanism for how IFNα improved APOBEC3 function without transcriptional induction remains to be determined. Surprisingly, both the potent (IFNα8) and weak (IFNα1) subtypes induced retroviral GG→AG hypermutation, suggesting that the deaminase-dependent activity of A3G did not correlate with the relative antiretroviral potencies of the IFNα subtypes. A3G inhibits HIV-1 through a deaminase-independent and deaminase-dependent mechanism. The deaminase-independent mechanism acts upstream by inhibiting the elongation of reverse transcripts, thereby preventing the production of single stranded DNA substrates for deamination [43]. Our results raise the intriguing possibility that IFNα subtypes may differentially activate deaminase-independent and deaminase-dependent activities of the APOBEC3 proteins. Notably, several studies suggested that A3G deaminase activity could be a double-edged sword, as A3G may not only restrict HIV-1 replication but also promote viral evolution to evade antiretroviral drugs and adaptive immunity [73–76]. About 16% of transmitted/founder HIV-1 strains exhibit signatures of G→A hypermutation [56], and APOBEC3-linked mutations in rapidly evolving sites may be linked to CTL escape [77]. Thus, the induction of weakly antiviral subtypes such as IFNα1 by pDCs during acute HIV-1 infection may have important consequences for early HIV-1 evolution.

In conclusion, the differential expression, potency and restriction factor induction by the human IFNα subtypes suggest that these evolutionarily related cytokines play non-redundant roles during HIV-1 infection. These findings are particularly timely with respect to ongoing clinical trials that aim to leverage IFNα2 therapy as a potential HIV-1 curative strategy (clinicaltrials.gov identifiers NCT00594880, NCT01295515, NCT01285050 and NCT01935089). Our results suggest that evaluating IFNα subtypes that more potently augmented APOBEC3-mediated deaminase-independent restriction may yield better clinical outcomes on the road to a functional HIV-1 cure.

## Materials and Methods

### Ethics statement

Blood collection from self-identified HIV-negative donors was approved by the Colorado Multiple Institutional Review Board (COMIRB) at the University of Colorado Anschutz Medical Campus. The use of discarded, macroscopically normal human jejunum tissue samples was granted exempt status by COMIRB and patients signed a pre-operative consent form allowing its unrestricted use for research purposes. Protected patient information was de-identified to laboratory personnel.

### Viral stocks

HIV-1BaL stocks (AIDS Research Reagent Program/ARRP Catalogue #4984) were prepared by passage in MOLT4-CCR5 (ARRP #510) cells for 9 days. Virus containing supernatants were ultracentrifuged at 76,800g. T/F HIV-1 infectious molecular clones CH470, CH40, and CH58, as well as AD17, CH106, CH607, REJO, RHPA, THRO, STCOr1, STCOr2, WARO, MCST, RHGA, TRJO and WITO were generously provided by Beatrice Hahn [57,58]. NL4-3 and NL4-3ΔVif were obtained from ARRP. CH470, CH40 and CH58 plasmids were re-transformed and amplified in Stbl3 cells (Invitrogen) and purified using Qiagen maxi kit. T/F maxi-preps were sequence-verified using 13 HIV-specific primers to cover the entire genome. 40 μg of T/F plasmids were used to transfect 293T cells in a T175 flask. Four flasks were transfected by CaCl2 transfection method for each virus [78]. Virus-containing supernatants were collected at 48 hrs, concentrated by ultracentrifugation at 76,800g over a 20% sucrose cushion. Virus stocks were titered using an HIV-1 Gag p24 ELISA kit (Perkin Elmer).

### Isolation and exposure of pDCs to HIV-1

pDCs were isolated from peripheral blood of 4 healthy donors who self-identified as HIV-1-uninfected. All subjects voluntarily gave written, informed consent. This study was approved by the Colorado Multiple Institutional Review Board (COMIRB) at the University of Colorado Anschutz Medical Campus. pDCs were negatively selected using the EasySep plasmacytoid cell enrichment kit according to the manufacturer’s instructions. Purity was determined by flow cytometry. On average, the pDC-enriched fraction was 76% (range: 53–92%) BDCA-2+. The other cell subpopulations were significantly depleted, with 2% CD3+ (from 65%), 0.2% CD14+ (from 6%), 0.7% CD19+ (from 2%) and 1.1% CD56+ (from 11%). Zombie Aqua Viability Dye (Biolegend) exclusion was used to identify viable cells, and anti-BDCA2-PE (Miltenyi) was used to identify pDCs. pDCs or PBMCs with pDCs removed were resuspended to 10^6^ cells/ml in complete RPMI (RPMI with 10% human AB serum, 1% penicillin/streptomycin/glutamine, 500 μg/ml Zosyn). Cells were spinoculated with 250 ng/ml of cell-free HIV-1_BaL_ for 2 hrs at 1700 rcf at room temperature. Cells were washed 1x with complete RPMI, resuspended to 10^6^ cells/ml, and incubated at 37°C for 4 hr. Cells were then harvested and RNA extracted using Qiagen RNAeasy Micro kit.

### Quantitative PCR for total IFNA transcripts

Primers were designed in conserved regions of the IFNA subtypes: Forward primer 5’TCCATGAGVTGATBCAGCAGA and reverse primer 5’ ATTTCTGCTCTGACAACCTCCC (S1A Fig). cDNA was transcribed from RNA using random hexamers in the Qiagen Quantitect Reverse Transcription kit. cDNA was diluted 1:5 and 10 μl added to make a final concentration of 1× Quantitect SYBR green PCR reagent containing 8 pmol of each primer. qPCR was run on Biorad CFX96 real-time PCR machine under the following conditions: 95°C for 15 min followed by 40 cycles of 94°C 15 s, 55°C 30 s, 72°C 30 s. Specificity was determined by melt curve analysis. qPCR data was analyzed with CFX Manager software (Biorad). Copy number was interpolated using a standard curve with 108–102 copies of IFNA8 plasmid. Copies of GAPDH were determined by Taqman primer/probe assay (S1 Table).

### IFNA subtype determination by Illumina sequencing

RNA from pDCs was reverse transcribed with Quantitect reverse-transcription kit (Qiagen) using a primer from a conserved region in the IFNA subtype alignment (S1A Fig). RT primer: 5’-GATCTCATGATTTCTGCTCTGAC. cDNA was added to a PCR reaction containing Phusion Hi Fidelity Taq (New England Biolabs) according to manufacturers instructions containing 8 pmol of the following Illumina primers containing random nucleotides (N) and 6-bp barcodes (INDEX#).

Forward primer: 5’AATGATACGGCGACCACCGAGATCTACACTCTTTCCCTACACGACGCTCTTCCGAT

CT NNNN INDEX1 TGCGTCTCCATGAGVTGATBCAGCAGA

Reverse primer: 5’CAAGCAGAAGACGGCATACGAGATGTGACTGGAGTTCAGACGTGTGCTCTTCCGATCT NNNN INDEX2 ATTTCTGCTCTGACAACCTCCC

PCR was run at the following conditions: 98°C for 30 min, followed by 35 cycles of 98°C 10 s, 58°C 15 s, 72°C 15 s, and a final elongation of 72°C 5 min. Sequences reads were generated in the Illumina MiSeq as recommended by the manufacturer. Resultant sequences were compared to a reference sequence database containing cDNA sequences from all members of *IFNA* gene family and identified as a particular *IFNA* subtype with a threshold of 90% identity. *IFNA* gene distribution was calculated based on a percentage of the total *IFNA* counts. *IFNA1* and *IFNA13* DNA sequences were identical in the amplified region and encode an identical protein and so were referred to as *IFNA1/13*. For simplicity the recombinant protein was noted as IFNα1.

### Recombinant IFNα subtypes

All 12 recombinant IFNα subtypes were purchased from PBL Assay Science, Cat. No. 11002–1. The proteins were resuspended in PBS containing 0.1% BSA to 5.31 μg/ml according to product insert and stored at –80°C as single use aliquots.

### LPMC collection and processing

Macroscopically normal human jejunum tissue samples were obtained from patients undergoing elective abdominal surgery. The use of discarded tissue was granted exempt status by COMIRB and patients signed a pre-operative consent form allowing its unrestricted use for research purposes. Protected patient information was de-identified to laboratory personnel. LPMCs were obtained and processed as previously described [32,33]. Briefly, LP mucosa was separated from muscularis mucosa, EDTA was used to separate epithelial cells, and collagenase D treatment released LPMCs. Cells were cryopreserved in RPMI + 10% DMSO + 10% FBS.

### HIV-1 infection of LPMCs

Cryopreserved LPMCs were thawed by gradual addition of thaw media (90 ml RPMI + 10% FBS + 1% penicillin/streptomycin/glutamine + 100 μl DNAse). LPMCs were resuspended to 2.5×10^6^ cells/ml in complete RPMI. HIV-1 (10 ng p24/ml for Ba-L and T/F HIV-1 strains) was added and spinoculated at 1700 rcf for 2 hr at room temperature. Cells were washed 1× in complete RPMI, resuspended, and plated onto V-bottom 96 well plates at a concentration of 10^6^ cells/ml. IFNα subtypes (PBL Assay Science) were added once at a final concentration of 100 pg/ml immediately post-infection. Cells were incubated for 4 days at 37°C, then were harvested at 4 dpi for flow cytometry as previously described [32,33]. Zombie Aqua Viability Dye (Biolegend) exclusion was used to identify viable cells. The antibodies used for flow cytometry were: CD3-ECD (Beckman Coulter) or CD3-PerCP-Cy5.5 (Tonbo Biosciences), CD8-APC (BD Pharmingen), HIV-1 p24-PE (Beckman Coulter). Data were collected on a Gallios 561 flow cytometer (Beckman Coulter) and analyzed using Kaluza version 1.2 (Beckman Coulter).

Supernatants were harvested at 4 dpi and infectious titer was determined in TZM.bl reporter cells. TZM.bl cells (1 x 10^4^) were plated in a 96-well plate in 160 μl culture media (DMEM + 10% FBS + 1% PSG) with dextran sulfate (100 ng/ml). 4 dpi supernatants (40 μl) were added directly to individual wells and incubated for 48 hrs at 37°C. Half of the media was removed and cells were lysed with 100 μl Britelite luciferase reagent (Perkin Elmer), incubated for at least 1 minute, and Relative Light Units (RLU) of luminescence were determined in a VictorX5 plate reader (Perkin Elmer). The supernatants were also titered using an HIV-1 Gag p24 ELISA kit (Perkin Elmer).

### ISG qPCR

Taqman primer probe combinations were used to quantify A3G, A3D, A3F, Tetherin and Mx2 relative to GAPDH (S1 Table). GeneExpression Mastermix (Life Technologies) was used according to instructions and contained 10 pmol of each primer and probe. Thermocycling conditions were as follows: 50°C 2 min and 95°C 10 min, then 40 cycles of 95°C 15 s and variable annealing temperatures (GAPDH: 64.5°C 45 s; Mx2: 62.5°C 45 s; BST-2: 60.8°C 45 s; and A3G: 56°C 40 s; A3F: 59°C 90 s; A3D: 60°C 45 s). Plates were run in the Biorad CFX96 real-time PCR machine.

### Mutation analysis of proviral HIV-1 DNA

Infection of LPMCs with HIV-1 T/F or NL4-3 virus stocks with or without IFNα treatment was performed as above. At 4 dpi, cell pellets were harvested and DNA extracted using Qiagen DNAEasy kit. Amplification of the gp41/nef region was performed by nested PCR assembled as Phusion Taq reaction according to manufacturer protocol containing 10 pmol of the following primers. External PCR: Forward 5’-TTGCTCTGGAAAACTCATYTGCAC; Reverse 5’-TCAGGGAAGTAGCCTTGTGTGT. Thermocycling conditions included 98°C for 30 min and 35 cycles of 98°C 10 s, 59.5°C 20 s, 72°C 35 s and final elongation at 72°C 7 min. Following preamplification, Phusion Taq nested PCR with MiSeq-configured primers was performed:

Forward: 5’ATGATACGGCGACCACCGAGATCTACACTCTTTCCCTACACGACGCTCTTCCGATCT NNNN INDEX1 AGCAGTAGCTGARGGRACAGAT

Reverse: 5’CAAGCAGAAGACGGCATACGAGATGTGACTGGAGTTCAGACGTGTGCTCTTCCGATCT NNNN INDEX2 AGTGAAYTARCCCTTCCAGTCC

with the following conditions: 98°C for 30 min, 35 cycles of 98°C 10 s, 56.6°C 15 s, 72°C 17 s and final elongation at 72°C 7 min. Amplicons were sequenced by Illumina MiSeq following standard protocol. Sequences with >80% identity were matched to the corresponding reference T/F HIV-1 sequences and total, GG→AG and GA→AA mutations were evaluated using custom Perl scripts [60,79].

### Statistical analysis

Data were analyzed using Prism 5.0 (GraphPad). For comparisons of data with over 2 variables (e.g., IFNα subtypes) obtained from the same donors (matched observations), repeated measures ANOVA was used for statistical analyses, followed a Dunnett’s multiple comparison test. For data with non-Gaussian distribution (evaluated using the Kolmogorov-Smirnov normality test), a nonparametric ANOVA using Friedman test was implemented followed by a Dunn’s posthoc pairwise analysis. For comparisons of two datasets, a two-tailed Student’s t-test was performed. Correlations between two datasets were determined by linear regression and evaluated by Pearson r. To compare the relative proportions of specific dinucleotide mutations, a 2 × 2 contingency analysis with Yates’ correction was used. For all statistical tests, *P* values < 0.05 were considered significant.

### Accession numbers

Next-generation sequencing data were deposited at the NCBI Sequence Archive Bioproject PRJNA284609. Accession numbers for IFNA genes used in this work are as follows. IFNA1, NM_024013.2; IFNA2, NM_000605.3; IFNA4, NM_021068.2; IFNA5, NM_002169.2; IFNA6, NM_021002.2; IFNA7, NM_021057.2; IFNA8, NM_002170.3; IFNA10, NM_002171.2; IFNA13, NM_006900.3; IFNA14, NM_002172.2; IFNA16, NM_002173.2; IFNA17, NM_021268.2; IFNA21, NM_002175.2.

## Supporting Information

S1 Fig Quantifying IFNα subtype expression. (A) Alignment of human *IFNA* genes with forward, reverse and reverse transcription (RT) primer sites indicated. Dots correspond to identical nucleotides to that of the consensus. Note that the forward primer contained degenerate bases at –8 and –13 positions (red) to capture the polymorphisms at these sites. (B) Validation of qPCR primers. Plasmids encoding *IFNA1*, *IFNA6*, *IFN14* and *IFNA8* were used as standards in a qPCR assay. Best-fit linear regression standard curves plotting cycle threshold with plasmid quantity (from 10^8^ to 10^2^ copies) were shown. Significant overlap between these standard curves suggested that the polymorphisms at the –8 and –13 positions in the forward primer did not result in variable efficiencies in amplifying diverse *IFNA* subtypes.
(TIF)

S2 Fig Induction of *IFNA* subtypes in pDCs following HIV-1 exposure. The absolute copy number for each *IFNA* subtype was computed by multiplying the percent distribution in Fig 1D and 1E with the total copies of *IFNA* in Fig 1B. Fold-induction per donor was computed by obtaining the ratio of *IFNA* subtype copy number in the pDC+HIV and pDC only condition. Error bars correspond to SEM from 4 pDC donors. All *IFNA* subtypes were induced in pDCs following HIV-1 exposure but the fold-induction ranged from 97.6-fold (*IFNA6*) to 1759-fold (*IFNA14*).
(TIF)

S3 Fig HIV-1 infection assays. HIV-1 infection levels were evaluated at 4 dpi, allowing for at least one round of HIV-1 replication. (1) Infectious titer in the supernatant was measured using the TZM.bl cells, a HeLa cell line expressing HIV-1 receptors and an HIV-1 LTR-driven luciferase promoter. Expression of HIV-1 Tat in TZM.bl cells leads to luciferase expression. The requirement for HIV-1 entry and Tat expression makes this a suitable assay to measure of infectious virus release. (2) Total virus particles was measured by ELISA for p24 capsid antigen. This assay would not distinguish between infectious and noninfectious particles. (3) Virion infectivity was measured by obtaining the ratio of infectious titer based on the TZM.bl assay and total virus particle release based on p24 ELISA. (4) HIV-1-infected cells were measured by intracellular p24 flow cytometry. Note that only infectious particles would infect CD4+ T cells in the next round of replication. Most HIV-1 restriction factors such as Mx2 and Tetherin act in the infected cell, resulting in a decrease in (2) total virus particle titers and (4) HIV-1 infected cells. Mx2 and Tetherin should also reduce (1) infectious titers in proportion to the total particles, and thus should not affect (3) virion infectivity. By contrast, members of the APOBEC3 family do not inhibit (2) total virus particle titers in the first round of replication, but inhibit HIV-1 replication in the next target cell. Thus, APOBEC3 activity should decrease (1) infectious titer, (3) virion infectivity and (4) HIV-1 infected cells by 4 dpi.
(TIF)

S4 Fig Correlation between IFNα antiviral potency based on the TZM.bl assay and various parameters. Antiviral potencies of each IFNα subtype was computed from Fig 1D and correlated with (A) antiviral potency using the intracellular p24 flow cytometry assay in Fig 1C; (B) absolute *IFNA* copy numbers in pDCs exposed to HIV-1; and binding affinity to (C) IFNAR-2 and (D) IFNAR-1 based on published data [22]. For all panels, Pearson correlation analyses were performed, with R^2^ values and *p*-values shown. Best-fit linear regression curves are shown in the correlation was significant (*p*<0.05) or trending (*p* = 0.05).
(TIF)

S5 Fig IFNα14 inhibits virion infectivity of multiple T/F HIV-1 strains LPAC model. Two LPMC donors were infected with T/F HIV-1 strains AD17, CH106, CH607, REJO, RHPA, THRO, STCOr1, STCOr2, WARO, MCST, RHGA, TRJO and WITO then treated with 100 pg/ml of IFNα14. Supernatants at 4 dpi were evaluated for infectious titer by TZM.bl assay and virus particle release using p24 ELISA. The ratio was used to compute the virion infectivity values. Each connected line corresponds to a T/F HIV-1 strain. Data were analyzed using a 2-tailed paired Student’s t-test. **, *p*<0.01; ***, *p*<0.001. Note that in majority of cases, the virion infectivities decreased post-IFNα14 treatment, with the exception of strain RHGA for donor 1 and strain STCOR2 for donor 2.
(TIF)

S6 Fig A3F and A3D expression in the presence of IFNα subtypes. LPMCs (n = 3 donors) were thawed and infected with HIV-1_BaL_, then treated with 100 pg/ml of weak (gray bars) and potent (black bars) IFNα subtypes. IFNα1, IFNα2, IFNα8 and IFNα14 shown simply as 1, 2, 8 and 14. After 24 hr, CD4+ T cells were negatively selected and RNA extracted for qPCR. A3F and A3D were quantified using Taqman qPCR normalized to GAPDH levels. Mean fold-induction values are shown for the 3 donors.
(TIF)

S1 Table Primers and probes for ISG quantification. (DOCX)
