# Supplementary figures and images for: Interferon-α Subtypes in an Ex Vivo Model of Acute HIV-1 Infection: Expression, Potency and Effector Mechanisms
Source: PLoS Pathog. 2015 Nov 3;11(11):e1005254. doi: 10.1371/journal.ppat.1005254 (PMC4631339; doi:10.1371/journal.ppat.1005254)

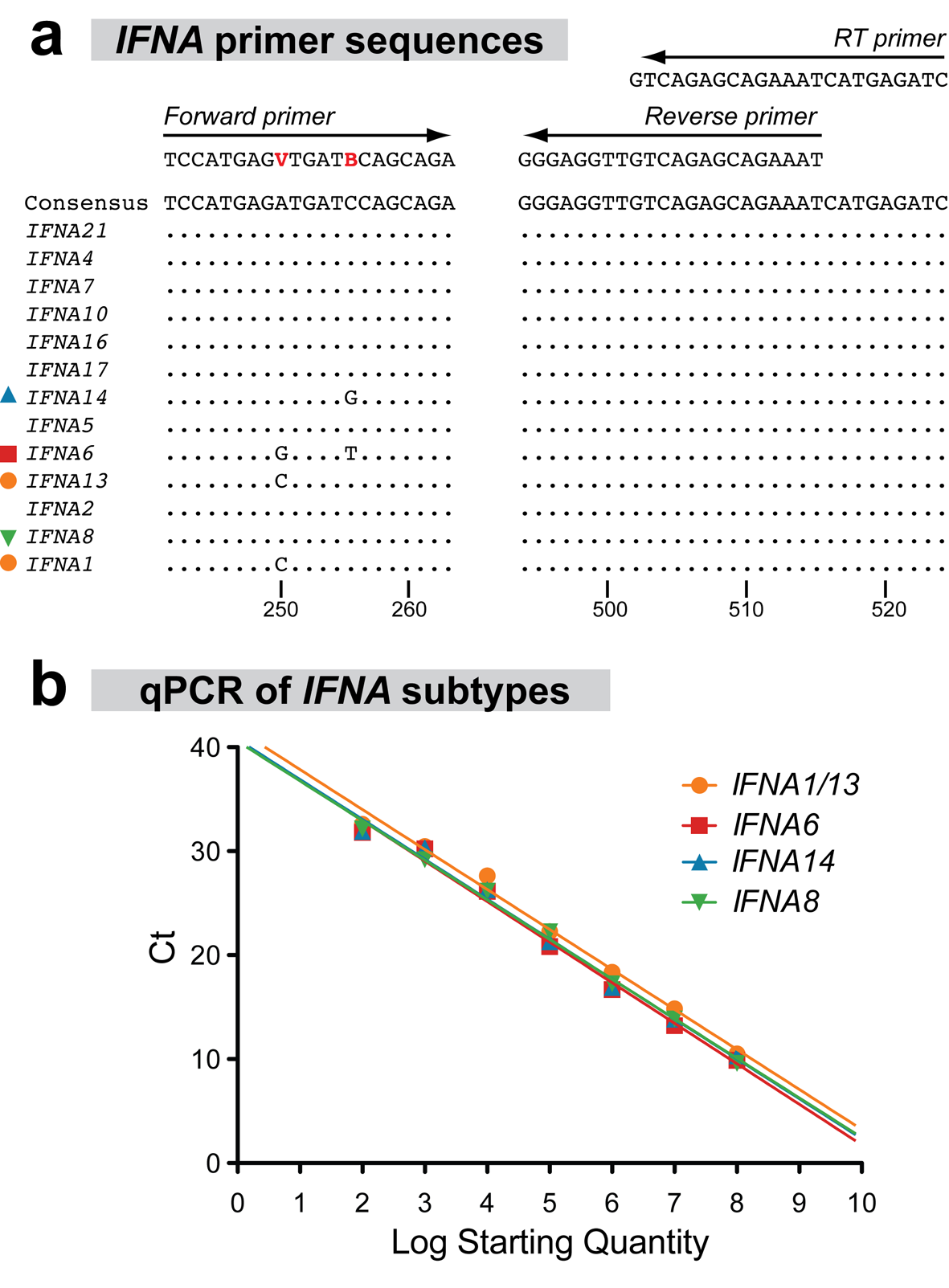

Supplement: S1 Fig — (A) Alignment of human IFNA genes with forward, reverse and reverse transcription (RT) primer sites indicated. Dots correspond to identical nucleotides to that of the consensus. Note that the forward primer contained degenerate bases at –8 and –13 positions (red) to capture the polymorphisms at these sites. (B) Validation of qPCR primers. Plasmids encoding IFNA1, IFNA6, IFN14 and IFNA8 were used as standards in a qPCR assay. Best-fit linear regression standard curves plotting cycle threshold with plasmid quantity (from 108 to 102 copies) were shown. Significant overlap between these standard curves suggested that the polymorphisms at the –8 and –13 positions in the forward primer did not result in variable efficiencies in amplifying diverse IFNA subtypes. (TIF) [file ppat.1005254.s001.tif]

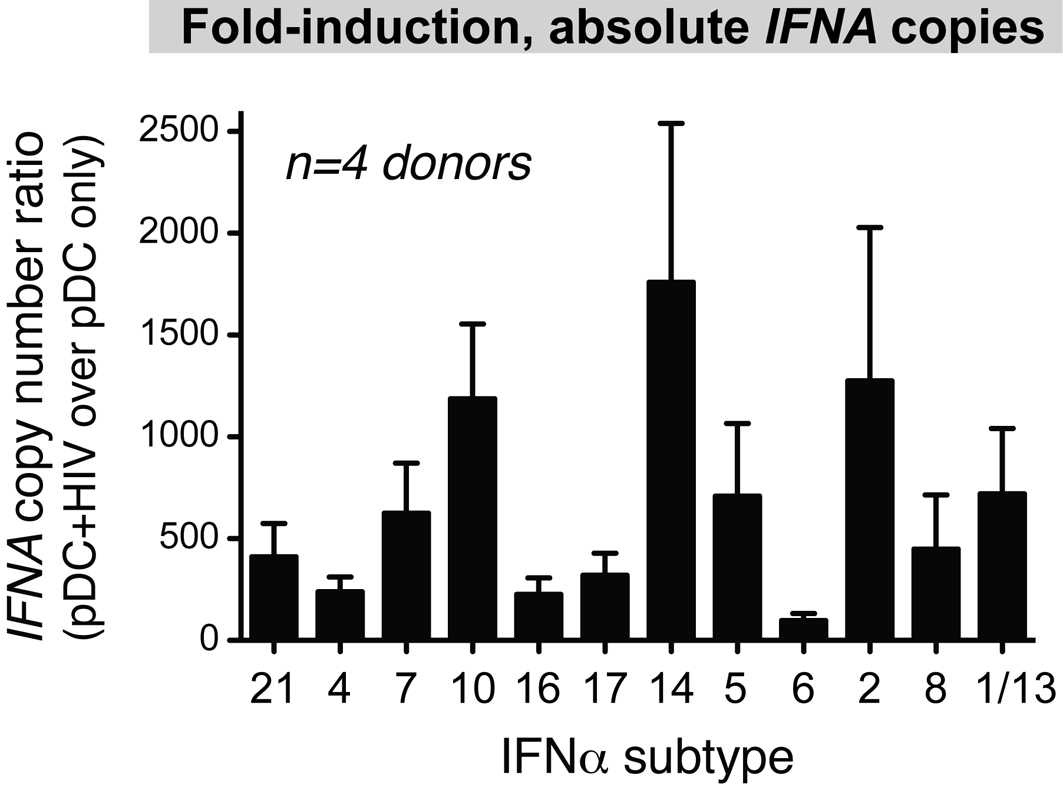

Supplement: S2 Fig — The absolute copy number for each IFNA subtype was computed by multiplying the percent distribution in Fig 1D and 1E with the total copies of IFNA in Fig 1B. Fold-induction per donor was computed by obtaining the ratio of IFNA subtype copy number in the pDC+HIV and pDC only condition. Error bars correspond to SEM from 4 pDC donors. All IFNA subtypes were induced in pDCs following HIV-1 exposure but the fold-induction ranged from 97.6-fold (IFNA6) to 1759-fold (IFNA14). (TIF) [file ppat.1005254.s002.tif]

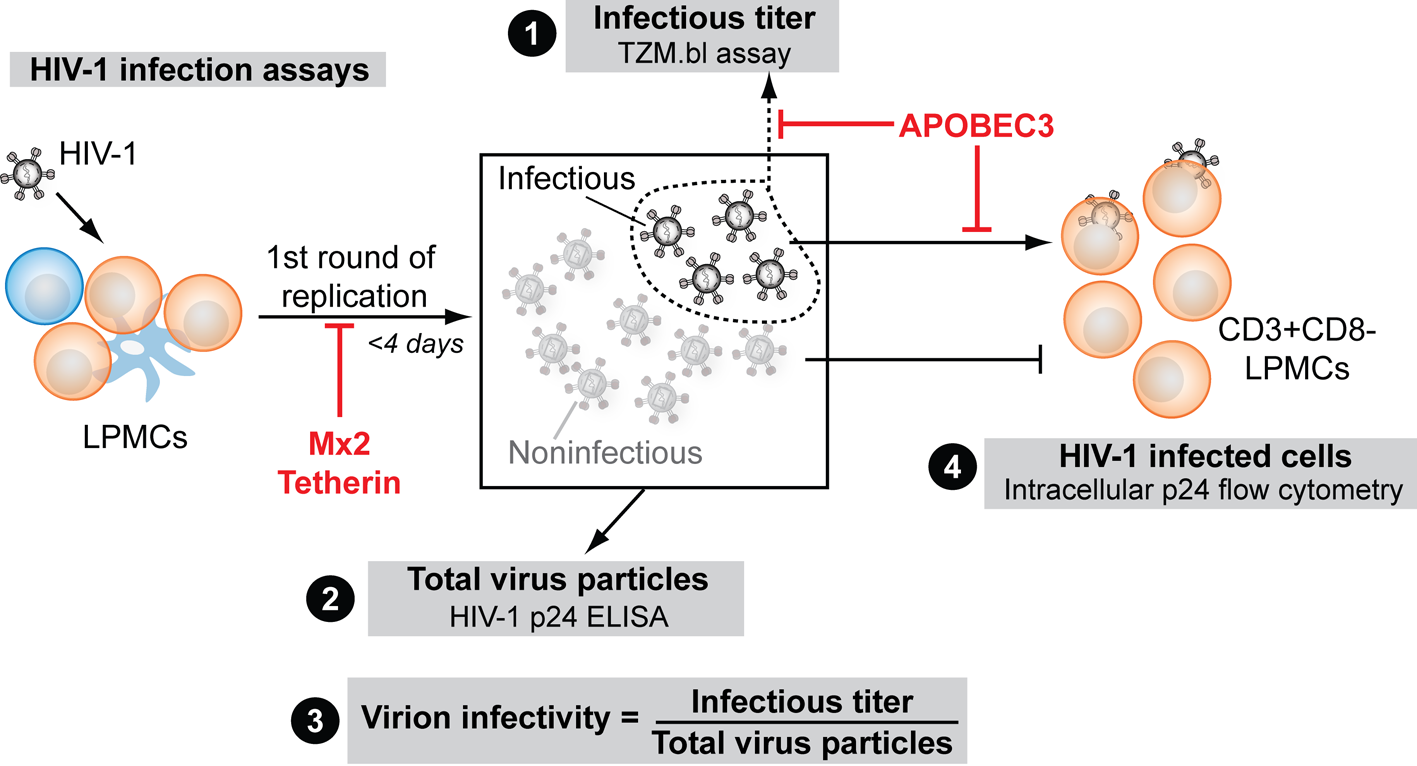

Supplement: S3 Fig — HIV-1 infection levels were evaluated at 4 dpi, allowing for at least one round of HIV-1 replication. (1) Infectious titer in the supernatant was measured using the TZM.bl cells, a HeLa cell line expressing HIV-1 receptors and an HIV-1 LTR-driven luciferase promoter. Expression of HIV-1 Tat in TZM.bl cells leads to luciferase expression. The requirement for HIV-1 entry and Tat expression makes this a suitable assay to measure of infectious virus release. (2) Total virus particles was measured by ELISA for p24 capsid antigen. This assay would not distinguish between infectious and noninfectious particles. (3) Virion infectivity was measured by obtaining the ratio of infectious titer based on the TZM.bl assay and total virus particle release based on p24 ELISA. (4) HIV-1-infected cells were measured by intracellular p24 flow cytometry. Note that only infectious particles would infect CD4+ T cells in the next round of replication. Most HIV-1 restriction factors such as Mx2 and Tetherin act in the infected cell, resulting in a decrease in (2) total virus particle titers and (4) HIV-1 infected cells. Mx2 and Tetherin should also reduce (1) infectious titers in proportion to the total particles, and thus should not affect (3) virion infectivity. By contrast, members of the APOBEC3 family do not inhibit (2) total virus particle titers in the first round of replication, but inhibit HIV-1 replication in the next target cell. Thus, APOBEC3 activity should decrease (1) infectious titer, (3) virion infectivity and (4) HIV-1 infected cells by 4 dpi. (TIF) [file ppat.1005254.s003.tif]

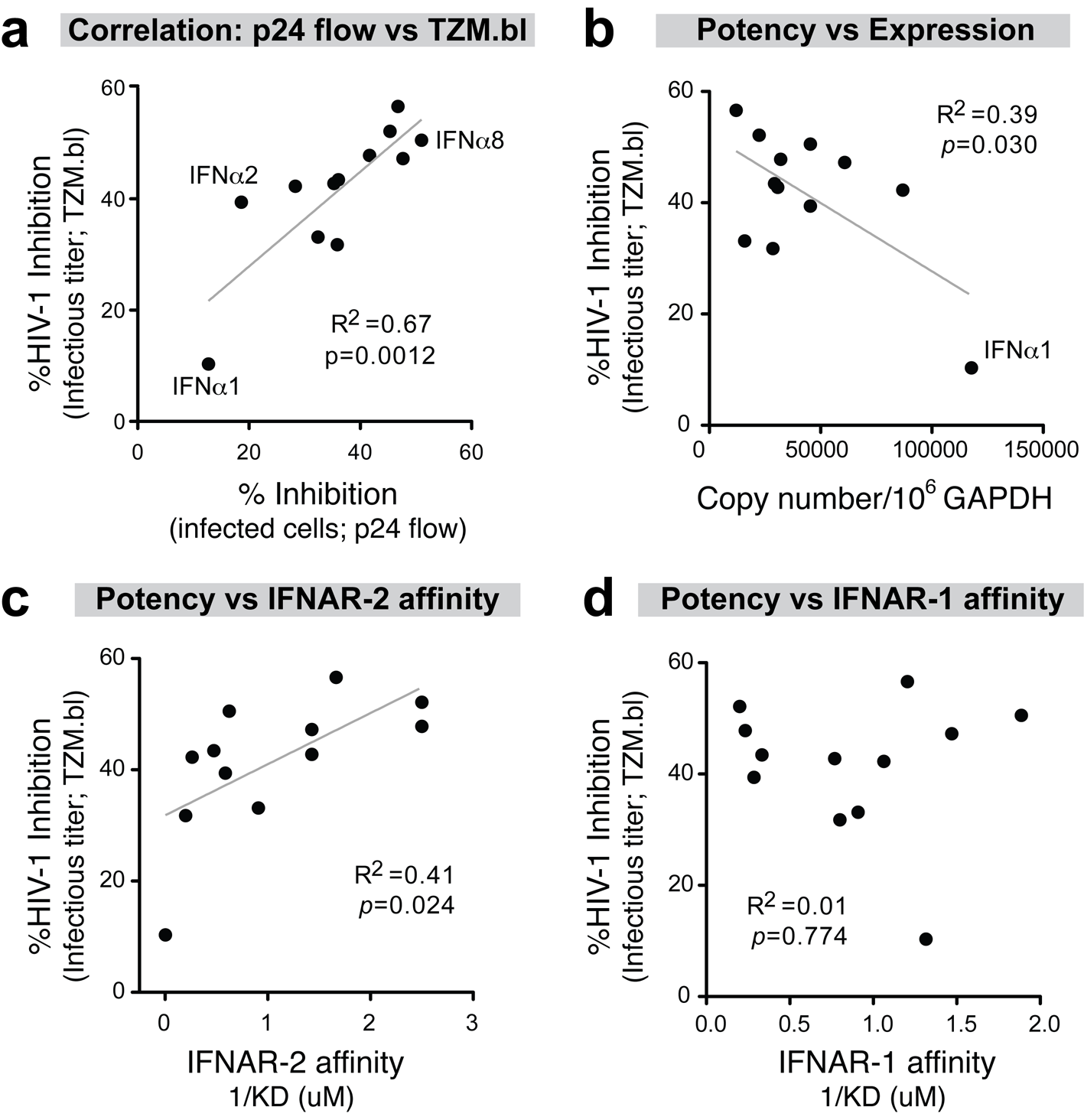

Supplement: S4 Fig — Antiviral potencies of each IFNα subtype was computed from Fig 1D and correlated with (A) antiviral potency using the intracellular p24 flow cytometry assay in Fig 1C; (B) absolute IFNA copy numbers in pDCs exposed to HIV-1; and binding affinity to (C) IFNAR-2 and (D) IFNAR-1 based on published data [22]. For all panels, Pearson correlation analyses were performed, with R2 values and p-values shown. Best-fit linear regression curves are shown in the correlation was significant (p<0.05) or trending (p = 0.05). (TIF) [file ppat.1005254.s004.tif]

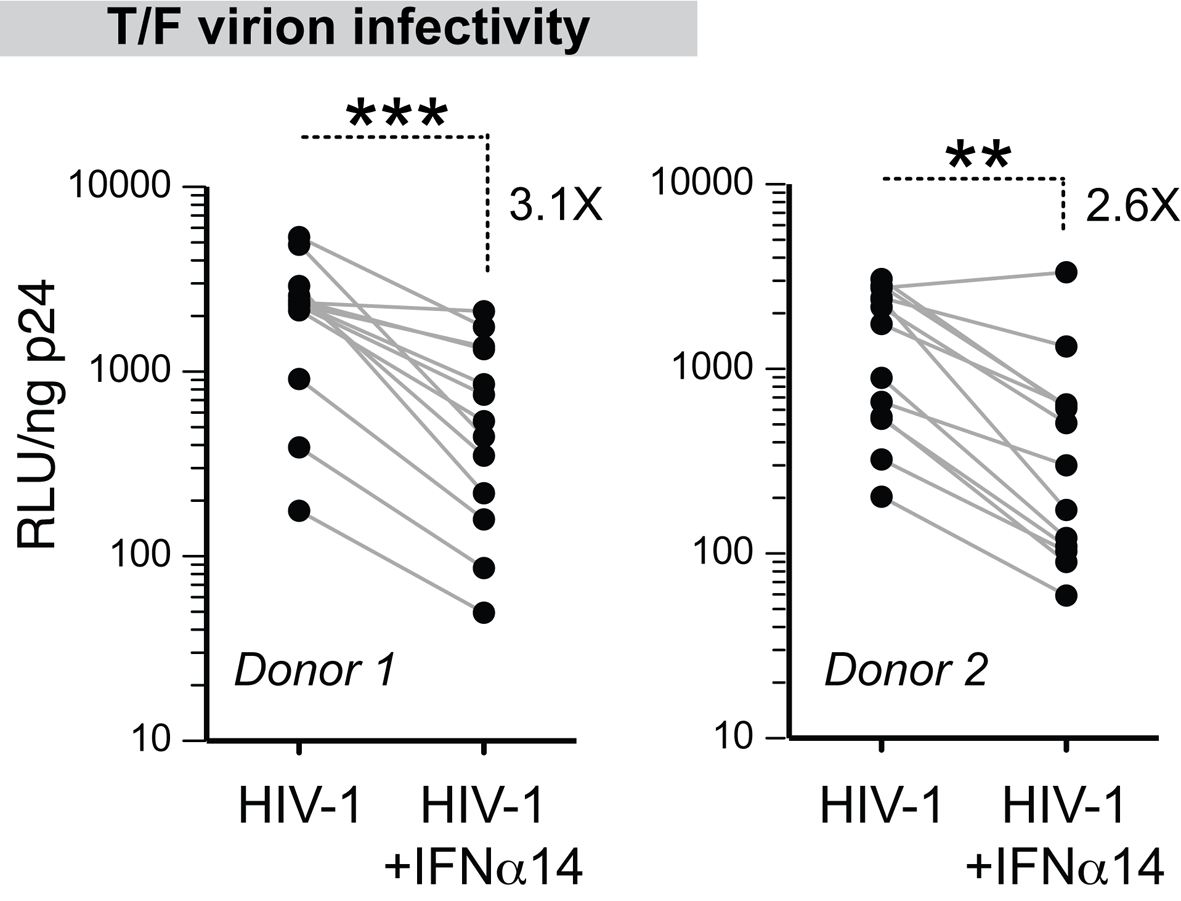

Supplement: S5 Fig — Two LPMC donors were infected with T/F HIV-1 strains AD17, CH106, CH607, REJO, RHPA, THRO, STCOr1, STCOr2, WARO, MCST, RHGA, TRJO and WITO then treated with 100 pg/ml of IFNα14. Supernatants at 4 dpi were evaluated for infectious titer by TZM.bl assay and virus particle release using p24 ELISA. The ratio was used to compute the virion infectivity values. Each connected line corresponds to a T/F HIV-1 strain. Data were analyzed using a 2-tailed paired Student’s t-test. **, p<0.01; ***, p<0.001. Note that in majority of cases, the virion infectivities decreased post-IFNα14 treatment, with the exception of strain RHGA for donor 1 and strain STCOR2 for donor 2. (TIF) [file ppat.1005254.s005.tif]

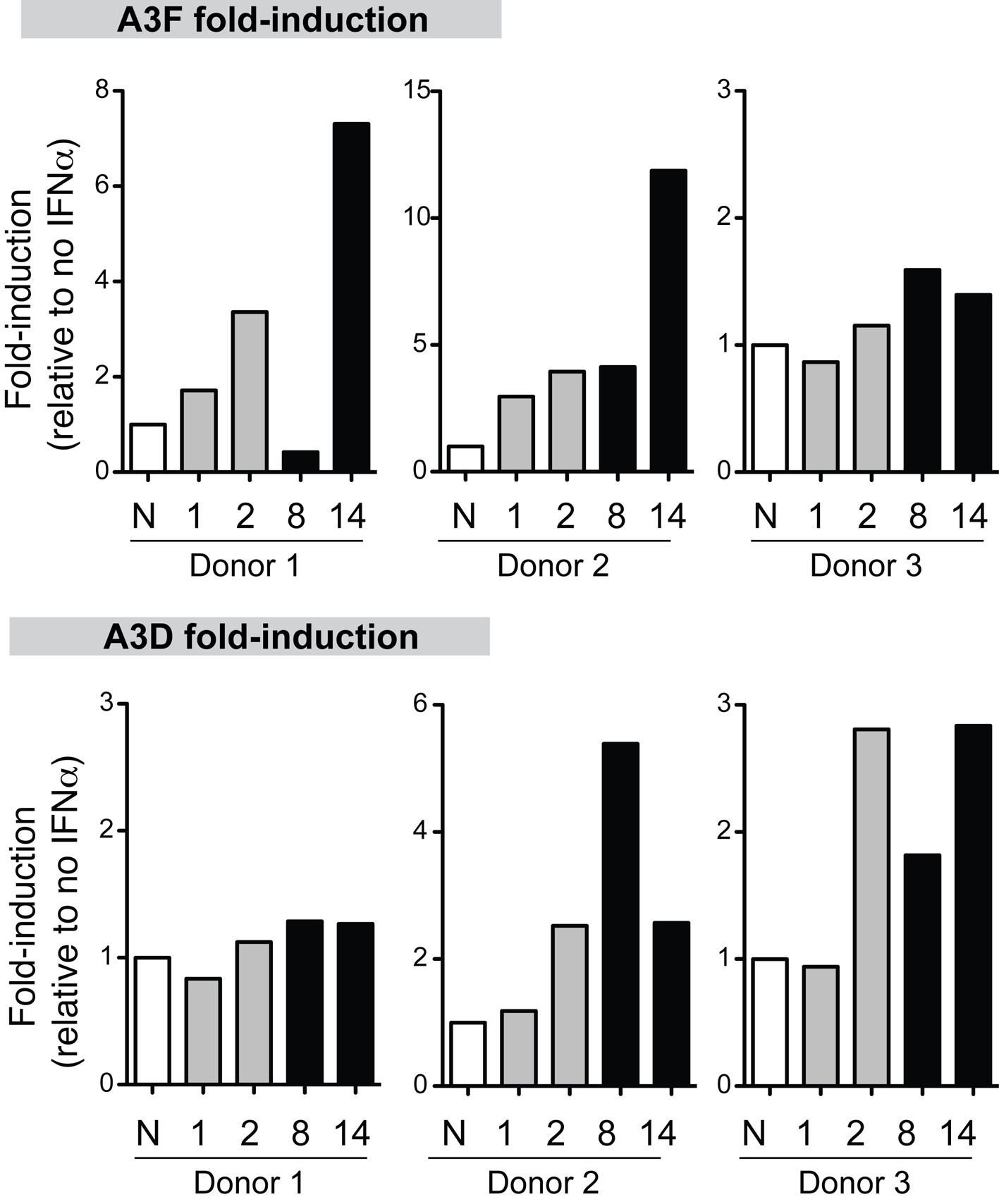

Supplement: S6 Fig — LPMCs (n = 3 donors) were thawed and infected with HIV-1BaL, then treated with 100 pg/ml of weak (gray bars) and potent (black bars) IFNα subtypes. IFNα1, IFNα2, IFNα8 and IFNα14 shown simply as 1, 2, 8 and 14. After 24 hr, CD4+ T cells were negatively selected and RNA extracted for qPCR. A3F and A3D were quantified using Taqman qPCR normalized to GAPDH levels. Mean fold-induction values are shown for the 3 donors. (TIF) [file ppat.1005254.s006.tif]
