# Supplementary material for: Interferon-α Subtypes in an Ex Vivo Model of Acute HIV-1 Infection: Expression, Potency and Effector Mechanisms
Source: PLoS Pathog. 2015 Nov 3;11(11):e1005254. doi: 10.1371/journal.ppat.1005254 (PMC4631339; doi:10.1371/journal.ppat.1005254)
Supplement: S1 Table — (DOCX) [file ppat.1005254.s007.docx]

**S1 Table.** **Primers and probes for ISG quantification.**

| Mx2.F | TTCCCAGTAATGAGTCTTCGGTTT |
| --- | --- |
| Mx2.R | AGTCACCATTCTCTCGGAGC |
| Mx2.P | [6~FAM]ACGTCTCGCCAACCAGATCCC[TAMRA~6~FAM] |
| BST2.F | TATGACTATTGCAGAGTGCCCATG |
| BST2.R | GTGACATTGCGACACTCCA |
| BST2.P | [6~FAM]CATCAAGGCCAACAGCGAGGC[TAMRA~6~FAM] |
| A3D.F | ACGACAACTTTGAAAACGAACCCAT |
| A3D.R | ACCTCCTGCCTGTGATTCGAC |
| A3D.P | [6~FAM] TTTCGAGGCCCGGTACTACCCAAACGTC [TAMRA~6~FAM] |
| A3F.F | CCGTTTGGACGCAAAGAT |
| A3F.R | CCAGGTGATCTGGAAACACTT |
| A3F.P | [6~FAM] TTTCGAGGCCAGGTGTATTC [TAMRA~6~FAM] |
| A3G.F | ACGGCATGAGACTTACCTGT |
| A3G.R | GGCCTTCAAGGAAACCGTGT |
| A3G.P | [6~FAM]CAGCGCAGGGGCTTTCTATGCAAC[TAMRA~6~FAM] |
| GAPDH.F | CCCATGTTCGTCATGGGTGT |
| GAPDH.R | TGGTCATGAGTCCTTCCACGAT |
| GAPDH.P | [6~FAM]CTGCACCACCAACTGCTTAGCACCC[TAMRA~6~FAM] |

Legend: F=forward primer; R=reverse primer; P=Taqman probe
